# Supplementary material for: Uncertainty and sensitivity analysis of the basic reproduction number of diphtheria: a case study of a Rohingya refugee camp in Bangladesh, November–December 2017
Source: PeerJ. 2018 Apr 2;6:e4583. doi: 10.7717/peerj.4583 (PMC5885970; doi:10.7717/peerj.4583)
Supplement: Supplemental Information 2 — Julia code of the estimation of the basic reproduction number. [file peerj-06-4583-s002.html]

20180226\_Rohignya in Julia


# Preamble¶

In [1]:

```
# load the functionality into Julia
using Plots, StatPlots
using ExcelReaders, FileIO, DataFrames, Query, Distributions, Optim
pyplot()
```

Out[1]:

```
Plots.PyPlotBackend()
```

# Loading the data¶

In [2]:

```
df = readxlsheet(DataFrame,"Data.xlsx",1) |>
    @query(i, begin
        @select {date=i.Date, i=i.Onset_Dec27}
        end) |> DataFrame
df[:i] = Int64.(df[:i]); df[:date] = DateTime.(df[:date]); df[:t] = 1:nrow(df);
df[:datestring] = Dates.format(df[:date], "u dd");
df |> head
```

Out[2]:

|  | date | i | t | datestring |
| --- | --- | --- | --- | --- |
| 1 | 2017-11-08T00:00:00 | 1 | 1 | Nov 08 |
| 2 | 2017-11-09T00:00:00 | 0 | 2 | Nov 09 |
| 3 | 2017-11-10T00:00:00 | 0 | 3 | Nov 10 |
| 4 | 2017-11-11T00:00:00 | 0 | 4 | Nov 11 |
| 5 | 2017-11-12T00:00:00 | 1 | 5 | Nov 12 |
| 6 | 2017-11-13T00:00:00 | 0 | 6 | Nov 13 |

In [3]:

```
# We omit last five datapoints due to the reporting reporting delay
df = df[maximum(df[:t])-df[:t].>=5,:]
```

Out[3]:

|  | date | i | t | datestring |
| --- | --- | --- | --- | --- |
| 1 | 2017-11-08T00:00:00 | 1 | 1 | Nov 08 |
| 2 | 2017-11-09T00:00:00 | 0 | 2 | Nov 09 |
| 3 | 2017-11-10T00:00:00 | 0 | 3 | Nov 10 |
| 4 | 2017-11-11T00:00:00 | 0 | 4 | Nov 11 |
| 5 | 2017-11-12T00:00:00 | 1 | 5 | Nov 12 |
| 6 | 2017-11-13T00:00:00 | 0 | 6 | Nov 13 |
| 7 | 2017-11-14T00:00:00 | 0 | 7 | Nov 14 |
| 8 | 2017-11-15T00:00:00 | 1 | 8 | Nov 15 |
| 9 | 2017-11-16T00:00:00 | 1 | 9 | Nov 16 |
| 10 | 2017-11-17T00:00:00 | 0 | 10 | Nov 17 |
| 11 | 2017-11-18T00:00:00 | 0 | 11 | Nov 18 |
| 12 | 2017-11-19T00:00:00 | 0 | 12 | Nov 19 |
| 13 | 2017-11-20T00:00:00 | 4 | 13 | Nov 20 |
| 14 | 2017-11-21T00:00:00 | 0 | 14 | Nov 21 |
| 15 | 2017-11-22T00:00:00 | 1 | 15 | Nov 22 |
| 16 | 2017-11-23T00:00:00 | 2 | 16 | Nov 23 |
| 17 | 2017-11-24T00:00:00 | 2 | 17 | Nov 24 |
| 18 | 2017-11-25T00:00:00 | 6 | 18 | Nov 25 |
| 19 | 2017-11-26T00:00:00 | 8 | 19 | Nov 26 |
| 20 | 2017-11-27T00:00:00 | 4 | 20 | Nov 27 |
| 21 | 2017-11-28T00:00:00 | 8 | 21 | Nov 28 |
| 22 | 2017-11-29T00:00:00 | 9 | 22 | Nov 29 |
| 23 | 2017-11-30T00:00:00 | 20 | 23 | Nov 30 |
| 24 | 2017-12-01T00:00:00 | 34 | 24 | Dec 01 |
| 25 | 2017-12-02T00:00:00 | 35 | 25 | Dec 02 |
| 26 | 2017-12-03T00:00:00 | 68 | 26 | Dec 03 |
| 27 | 2017-12-04T00:00:00 | 49 | 27 | Dec 04 |
| 28 | 2017-12-05T00:00:00 | 80 | 28 | Dec 05 |
| 29 | 2017-12-06T00:00:00 | 107 | 29 | Dec 06 |
| 30 | 2017-12-07T00:00:00 | 124 | 30 | Dec 07 |
| ⋮ | ⋮ | ⋮ | ⋮ | ⋮ |

In [4]:

```
@df df bar(:datestring,:i,legend=false, xrotation=60)
```

Out[4]:

# Generation time¶

(We assume that no infection before the onset of symptoms, so **generation time = serial interval**)

In [5]:

```
mean_gt = 8.0; # mean = 8 days
CV_gt = 0.5; #CV is 50% 

αgt=1/CV_gt^2; θgt = mean_gt/αgt

gt = Gamma(αgt,θgt)
show(gt)

plot(gt, fill=(0, .5, :orange), size=(350,220), legend=false, xlim=(0,32), xlab="day")
```

```
Distributions.Gamma{Float64}(α=4.0, θ=2.0)
```

Out[5]:

In [6]:

```
gen_time = (x -> cdf(gt,x)-cdf(gt,x-1)).(1:nrow(df))
plot(gen_time,size=(400,300),legend=false)
```

Out[6]:

In [7]:

```
quantile.(gt,[.025,.5,.975])
```

Out[7]:

```
3-element Array{Float64,1}:
  2.17973
  7.34412
 17.5345
```

# Latin hypercube sampling¶

In [8]:

```
## The function is adopted from:
## from https://github.com/robertfeldt/BlackBoxOptim.jl/blob/master/src/utilities/latin_hypercube_sampling.jl
"""
    latin_hypercube_sampling(mins, maxs, numSamples)
    Randomly sample `numSamples` values from the parallelogram defined
    by `mins` and `maxs` using the Latin hypercube algorithm.
"""
function latin_hypercube_sampling{T}(mins::AbstractVector{T}, maxs::AbstractVector{T}, numSamples::Integer)
    dims = length(mins)
    result = zeros(T, numSamples, dims)
    @inbounds for i in 1:dims
        interval_len = (maxs[i] - mins[i]) / numSamples
        result[:,i] = shuffle!(linspace(mins[i], maxs[i] - interval_len, numSamples) +
                               interval_len*rand(numSamples))
    end
    return result'
end
```

Out[8]:

```
latin_hypercube_sampling
```

In [9]:

```
nsamples = 1000

# for reproducibility we fix the seed of the random number generator
srand(7)
smpls = latin_hypercube_sampling([0.0],[1.0],nsamples);
```

### Vaccination coverage¶

- Symmetric triangular distribution

In [10]:

```
vmin = 0.0
vmax = 0.70
vpeak = (vmax-vmin)/2

vs = quantile.(TriangularDist(vmin,vmax,vpeak),smpls[1,:])
plot(TriangularDist(vmin,vmax,vpeak),legend=false,fill=(0,.5,:orange),size=(300,220),
    xlim=(0,1),xlab="v",ylab="probability density")
```

Out[10]:

In [11]:

```
plot(vs,legend=false)
hline!([vmin,vmax,vpeak],line=(:dash, :green),size=(450,300),xlab="# sample",ylab="v")
```

Out[11]:

# Log-likelihood function¶

In [12]:

```
N = 579384
println("Effective population size: $(Int64(N))")
```

```
Effective population size: 579384
```

In [13]:

```
v = (vmax+vmin)/2
kmax = nrow(df)
function getNegativeLoglk(params)
    # Log-exponential transformation of input variables to be sure that they all stay positive for the optimization routine
    R0 = exp(params[1])
    α1 = exp(params[2])
    α2 = exp(params[3])
    α = [[α1 for x in 1:34]; [α2 for x in 35:kmax]]
    loglk = 0
    for k in 2:kmax
        λ = R0*(1-v-sum(df[1:(k-1),:i]./α[1:(k-1)])/N)*α[k]*sum(df[1:(k-1),:i]./α[1:(k-1)].*gen_time[(k-1):-1:1])
        loglk += λ<=0 ? 0 : df[k,:i]*log(λ)-λ
    end
    return(-loglk)
end

getNegativeLoglk(log.([7,.1,.01]))
```

Out[13]:

```
-7565.503589993599
```

In [14]:

```
res0 = optimize(getNegativeLoglk, log.([6,0.1,0.1]))
```

Out[14]:

```
Results of Optimization Algorithm
 * Algorithm: Nelder-Mead
 * Starting Point: [1.791759469228055,-2.3025850929940455, ...]
 * Minimizer: [1.9681226141322927,-4.588687821908185, ...]
 * Minimum: -8.073713e+03
 * Iterations: 112
 * Convergence: true
   *  √(Σ(yᵢ-ȳ)²)/n < 1.0e-08: true
   * Reached Maximum Number of Iterations: false
 * Objective Calls: 209
```

In [15]:

```
res = Optim.minimizer(res0)
exp.(res)
```

Out[15]:

```
3-element Array{Float64,1}:
 7.15723  
 0.0101662
 0.0052803
```

### A bit modified function that incorporates some analytical derivation¶

(We can derive directly $R\_0$ as the function $\alpha\_1$, $\alpha\_2$ and $v$)

In [16]:

```
v = (vmax+vmin)/2
kmax = nrow(df)
function getNegativeLoglk(params) 
    α1 = exp(params[1])
    α2 = exp(params[2])
    α = [[α1 for x in 1:34]; [α2 for x in 35:kmax]]
    R0numerator = 0.0; R0denominator = 0.0
    for k in 2:kmax
        R0numerator += df[k,:i]
        R0denominator += (1-v-sum(df[1:(k-1),:i]./α[1:(k-1)])/N)*α[k]*sum(df[1:(k-1),:i]./α[1:(k-1)].*gen_time[(k-1):-1:1])
    end
    R0 = R0numerator/R0denominator
    loglk = 0
    for k in 2:kmax
        λ = R0*(1-v-sum(df[1:(k-1),:i]./α[1:(k-1)])/N)*α[k]*sum(df[1:(k-1),:i]./α[1:(k-1)].*gen_time[(k-1):-1:1])
        loglk += λ<=0 ? 0 : df[k,:i]*log(λ)-λ
    end
    return(-loglk)
end

function getR0(params) 
    α1 = exp(params[1])
    α2 = exp(params[2])
    α = [[α1 for x in 1:34]; [α2 for x in 35:kmax]]
    R0numerator = 0.0; R0denominator = 0.0;
    for k in 2:kmax
        R0numerator += df[k,:i]
        R0denominator += (1-v-sum(df[1:(k-1),:i]./α[1:(k-1)])/N)*α[k]*sum(df[1:(k-1),:i]./α[1:(k-1)].*gen_time[(k-1):-1:1])
    end
    
    return(R0numerator/R0denominator)
end

getNegativeLoglk(log.([.1,.1]))
```

Out[16]:

```
-7598.986560896426
```

In [17]:

```
res0 = optimize(getNegativeLoglk, log.([.01,.01]))
```

Out[17]:

```
Results of Optimization Algorithm
 * Algorithm: Nelder-Mead
 * Starting Point: [-4.605170185988091,-4.605170185988091]
 * Minimizer: [-4.5886861319200305,-5.243772128532607]
 * Minimum: -8.073713e+03
 * Iterations: 45
 * Convergence: true
   *  √(Σ(yᵢ-ȳ)²)/n < 1.0e-08: true
   * Reached Maximum Number of Iterations: false
 * Objective Calls: 88
```

In [18]:

```
res = Optim.minimizer(res0)
exp.(res)
```

Out[18]:

```
2-element Array{Float64,1}:
 0.0101662
 0.0052803
```

In [19]:

```
R0 = getR0(res)
```

Out[19]:

```
7.157230132177564
```

Testing that BFGS() method gives the same result as we just had for Nelder-Mead

In [20]:

```
res0 = optimize(getNegativeLoglk, log.([.01,.01]), BFGS())
```

Out[20]:

```
Results of Optimization Algorithm
 * Algorithm: BFGS
 * Starting Point: [-4.605170185988091,-4.605170185988091]
 * Minimizer: [-4.588686035118337,-5.243772440547911]
 * Minimum: -8.073713e+03
 * Iterations: 9
 * Convergence: false
   * |x - x'| ≤ 1.0e-32: false 
     |x - x'| = 9.11e-12 
   * |f(x) - f(x')| ≤ 1.0e-32 |f(x)|: false
     |f(x) - f(x')| = -1.13e-16 |f(x)|
   * |g(x)| ≤ 1.0e-08: false 
     |g(x)| = 1.64e-08 
   * Stopped by an increasing objective: true
   * Reached Maximum Number of Iterations: false
 * Objective Calls: 37
 * Gradient Calls: 37
```

# Uncertainty analysis¶

In [ ]:

```
R0s = Vector(nsamples)
α1s = Vector(nsamples)
α2s = Vector(nsamples)
initial = [.01, .01]
for k in 1:nsamples
    v = vs[k]
    println(k)
    res0 = optimize(getNegativeLoglk,log.(initial))
    res = Optim.minimizer(res0)
    α = [[exp(res[1]) for x in 1:34]; [exp(res[2]) for x in 35:kmax]]
    if (1-v-sum(df[:i]./α)/N)<=0 
        R0s[k] = -1; α1s[k] = -1; α2s[k] = -1
    else
        R0s[k] = getR0(res)
        α1s[k] = exp(res[1])
        α2s[k] = exp(res[2])
    end
end
```

### Results: statistics¶

In [22]:

```
dfResults = DataFrame(v = Float64.(vs), R0 = Float64.(R0s), α2 = Float64.(α2s), α1 = Float64.(α1s))
dfResults |> head
```

Out[22]:

|  | v | R0 | α2 | α1 |
| --- | --- | --- | --- | --- |
| 1 | 0.5894227250213706 | 11.330879490025549 | 0.008359434026414361 | 0.01609451073570251 |
| 2 | 0.10538829771244006 | 5.200247755484328 | 0.0038365168855488906 | 0.00738649045719166 |
| 3 | 0.43856148311409776 | 8.286219135812246 | 0.006113211293208447 | 0.01176984997305611 |
| 4 | 0.28102459847171557 | 6.4705981221981 | 0.00477373139842367 | 0.00919089373478796 |
| 5 | 0.32559869409401515 | 6.898279006193839 | 0.005089235539785856 | 0.009798399124634557 |
| 6 | 0.212055019837026 | 5.904217548090001 | 0.0043558793797612285 | 0.008386464300728069 |

In [23]:

```
describe(dfResults)
```

```
v
Summary Stats:
Mean:           0.350003
Minimum:        0.014108
1st Quartile:   0.247769
Median:         0.349980
3rd Quartile:   0.452274
Maximum:        0.685613
Length:         1000
Type:           Float64
Number Missing: 0
% Missing:      0.000000

R0
Summary Stats:
Mean:           7.550796
Minimum:        4.718778
1st Quartile:   6.184553
Median:         7.157012
3rd Quartile:   8.493670
Maximum:        14.797706
Length:         1000
Type:           Float64
Number Missing: 0
% Missing:      0.000000

α2
Summary Stats:
Mean:           0.005571
Minimum:        0.003481
1st Quartile:   0.004563
Median:         0.005280
3rd Quartile:   0.006266
Maximum:        0.010917
Length:         1000
Type:           Float64
Number Missing: 0
% Missing:      0.000000

α1
Summary Stats:
Mean:           0.010725
Minimum:        0.006703
1st Quartile:   0.008785
Median:         0.010166
3rd Quartile:   0.012064
Maximum:        0.021019
Length:         1000
Type:           Float64
Number Missing: 0
% Missing:      0.000000
```

In [24]:

```
mode(sort(dfResults,cols = [:R0])[:R0])
```

Out[24]:

```
4.718778020264982
```

In [25]:

```
describe(sort(dfResults,cols = [:R0])[26:(end-25),:R0])
```

```
Summary Stats:
Mean:           7.470383
Minimum:        5.047761
1st Quartile:   6.232535
Median:         7.157012
3rd Quartile:   8.401773
Maximum:        12.254293
Length:         950
Type:           Float64
Number Missing: 0
% Missing:      0.000000
```

In [26]:

```
describe(sort(dfResults,cols = [:α1])[26:(end-25),:α1])
```

```
Summary Stats:
Mean:           0.010611
Minimum:        0.007170
1st Quartile:   0.008853
Median:         0.010166
3rd Quartile:   0.011934
Maximum:        0.017406
Length:         950
Type:           Float64
Number Missing: 0
% Missing:      0.000000
```

In [27]:

```
describe(sort(dfResults,cols = [:α2])[26:(end-25),:α2])
```

```
Summary Stats:
Mean:           0.005511
Minimum:        0.003724
1st Quartile:   0.004598
Median:         0.005280
3rd Quartile:   0.006198
Maximum:        0.009041
Length:         950
Type:           Float64
Number Missing: 0
% Missing:      0.000000
```

### Results: Plots¶

In [28]:

```
scatter(α1s,R0s,xlab="α1",ylab="R0",legend=false,xlim=(0,.025))
```

Out[28]:

In [29]:

```
scatter(vs[1:nsamples],R0s,xlab="v",ylab="R0",legend=false,xlim=(0,.7))
```

Out[29]:

In [30]:

```
scatter(vs[1:nsamples],α1s,xlab="v",ylab="α1",legend=false,xlim=(0,.7),ylim=(0,.025))
```

Out[30]:

In [31]:

```
scatter(α2s,α1s,xlab="α2",ylab="α1",legend=false,xlim=(0,.025),ylim=(0,.025))
```

Out[31]:

In [32]:

```
histogram(R0s,legend=:none,xlab="R0")
```

Out[32]:

In [33]:

```
histogram(α1s,legend=:none,xlab="α1")
```

Out[33]:

In [34]:

```
histogram(α2s,legend=:none,xlab="α2")
```

Out[34]:

### Results: Output for plotting in R¶

In [35]:

```
writetable("output_Julia_50.csv",DataFrame(R0=R0s,a1=α1s,a2=α2s,v=vs))
```

# Sensitivity analysis for CV of the gen.time (Fig 4)¶

# mean = 8 days, CV = 25%¶

In [36]:

```
mean_gt = 8.0; # mean = 5 days
CV_gt = 0.25; #CV is 25% 

αgt=1/CV_gt^2; θgt = mean_gt/αgt;

gt = Gamma(αgt,θgt)
show(gt)

plot(gt, fill=(0, .5, :orange), size=(350,220), legend=false, xlim=(0,32), xlab="day")
```

```
Distributions.Gamma{Float64}(α=16.0, θ=0.5)
```

Out[36]:

In [37]:

```
gen_time = (x -> cdf(gt,x)-cdf(gt,x-1)).(1:nrow(df))
plot(gen_time,size=(400,300),legend=false)
```

Out[37]:

In [38]:

```
quantile.(gt,[.025,.5,.975])
```

Out[38]:

```
3-element Array{Float64,1}:
  4.57269
  7.83396
 12.3701
```

In [ ]:

```
R0s = Vector(nsamples)
α1s = Vector(nsamples)
α2s = Vector(nsamples)
initial = [.01, .01]
for k in 1:nsamples
    v = vs[k]
    println(k)
    res0 = optimize(getNegativeLoglk,log.(initial))
    res = Optim.minimizer(res0)
    α = [[exp(res[1]) for x in 1:34]; [exp(res[2]) for x in 35:kmax]]
    if (1-v-sum(df[:i]./α)/N)<=0 
        R0s[k] = -1; α1s[k] = -1; α2s[k] = -1
    else
        R0s[k] = getR0(res)
        α1s[k] = exp(res[1])
        α2s[k] = exp(res[2])
    end
end
```

### Results¶

In [40]:

```
dfResults = DataFrame(v = Float64.(vs), R0 = Float64.(R0s), α2 = Float64.(α2s), α1 = Float64.(α1s))
dfResults |> head
```

Out[40]:

|  | v | R0 | α2 | α1 |
| --- | --- | --- | --- | --- |
| 1 | 0.5894227250213706 | 14.830832768661402 | 0.007299017478872421 | 0.01898319181431099 |
| 2 | 0.10538829771244006 | 6.806535395452376 | 0.0033498421082325414 | 0.00871229613062067 |
| 3 | 0.43856148311409776 | 10.845709966334601 | 0.005337738674817823 | 0.013882325034331747 |
| 4 | 0.28102459847171557 | 8.469270813378724 | 0.004168171333606691 | 0.01084053973340195 |
| 5 | 0.32559869409401515 | 9.029042302409472 | 0.004443662278249169 | 0.01155708479137852 |
| 6 | 0.212055019837026 | 7.727957015362263 | 0.003803322214579971 | 0.009891700323493829 |

In [41]:

```
describe(dfResults)
```

```
v
Summary Stats:
Mean:           0.350003
Minimum:        0.014108
1st Quartile:   0.247769
Median:         0.349980
3rd Quartile:   0.452274
Maximum:        0.685613
Length:         1000
Type:           Float64
Number Missing: 0
% Missing:      0.000000

R0
Summary Stats:
Mean:           9.883128
Minimum:        6.176348
1st Quartile:   8.094852
Median:         9.367707
3rd Quartile:   11.117247
Maximum:        19.368472
Length:         1000
Type:           Float64
Number Missing: 0
% Missing:      0.000000

α2
Summary Stats:
Mean:           0.004864
Minimum:        0.003040
1st Quartile:   0.003984
Median:         0.004610
3rd Quartile:   0.005471
Maximum:        0.009532
Length:         1000
Type:           Float64
Number Missing: 0
% Missing:      0.000000

α1
Summary Stats:
Mean:           0.012650
Minimum:        0.007906
1st Quartile:   0.010361
Median:         0.011991
3rd Quartile:   0.014230
Maximum:        0.024791
Length:         1000
Type:           Float64
Number Missing: 0
% Missing:      0.000000
```

In [42]:

```
mode(sort(dfResults,cols = [:R0])[:R0])
```

Out[42]:

```
6.176347535689983
```

In [43]:

```
describe(sort(dfResults,cols = [:R0])[26:(end-25),:R0])
```

```
Summary Stats:
Mean:           9.777877
Minimum:        6.606941
1st Quartile:   8.157666
Median:         9.367707
3rd Quartile:   10.996969
Maximum:        16.039505
Length:         950
Type:           Float64
Number Missing: 0
% Missing:      0.000000
```

### Results: Plots¶

In [44]:

```
histogram(R0s,legend=:none,xlab="R0")
```

Out[44]:

### Results: Output for plotting in R¶

In [45]:

```
writetable("output_Julia_25.csv",DataFrame(R0=R0s,a1=α1s,a2=α2s,v=vs))
```

# mean = 8 days, CV = 75%¶

In [46]:

```
mean_gt = 8.0; # mean = 5 days
CV_gt = 0.75; #CV is 75% 

αgt=1/CV_gt^2; θgt = mean_gt/αgt;

gt = Gamma(αgt,θgt)
show(gt)

plot(gt, fill=(0, .5, :orange), size=(350,220), legend=false, xlim=(0,32), xlab="day")
```

```
Distributions.Gamma{Float64}(α=1.7777777777777777, θ=4.5)
```

Out[46]:

In [47]:

```
gen_time = (x -> cdf(gt,x)-cdf(gt,x-1)).(1:nrow(df))
plot(gen_time,size=(400,300),legend=false)
```

Out[47]:

In [48]:

```
quantile.(gt,[.025,.5,.975])
```

Out[48]:

```
3-element Array{Float64,1}:
  0.79649
  6.56022
 23.3133
```

In [ ]:

```
R0s = Vector(nsamples)
α1s = Vector(nsamples)
α2s = Vector(nsamples)
initial = [.01, .01]
for k in 1:nsamples
    v = vs[k]
    println(k)
    res0 = optimize(getNegativeLoglk,log.(initial))
    res = Optim.minimizer(res0)
    α = [[exp(res[1]) for x in 1:34]; [exp(res[2]) for x in 35:kmax]]
    if (1-v-sum(df[:i]./α)/N)<=0 
        R0s[k] = -1; α1s[k] = -1; α2s[k] = -1
    else
        R0s[k] = getR0(res)
        α1s[k] = exp(res[1])
        α2s[k] = exp(res[2])
    end
end
```

### Results¶

In [50]:

```
dfResults = DataFrame(v = Float64.(vs), R0 = Float64.(R0s), α2 = Float64.(α2s), α1 = Float64.(α1s))
dfResults |> head
```

Out[50]:

|  | v | R0 | α2 | α1 |
| --- | --- | --- | --- | --- |
| 1 | 0.5894227250213706 | 8.971262436309477 | 0.0097584885165203 | 0.01438885249492456 |
| 2 | 0.10538829771244006 | 4.1173085105978275 | 0.00447861907355831 | 0.006603657486849202 |
| 3 | 0.43856148311409776 | 6.560648427733782 | 0.007136336579460204 | 0.01052243463987549 |
| 4 | 0.28102459847171557 | 5.123123291197905 | 0.0055726584696024085 | 0.008216913831927707 |
| 5 | 0.32559869409401515 | 5.4617261615206845 | 0.005941005695949644 | 0.008759921575527227 |
| 6 | 0.212055019837026 | 4.674678071276846 | 0.005084901400846277 | 0.007497664521506781 |

In [51]:

```
describe(dfResults)
```

```
v
Summary Stats:
Mean:           0.350003
Minimum:        0.014108
1st Quartile:   0.247769
Median:         0.349980
3rd Quartile:   0.452274
Maximum:        0.685613
Length:         1000
Type:           Float64
Number Missing: 0
% Missing:      0.000000

R0
Summary Stats:
Mean:           5.978367
Minimum:        3.736111
1st Quartile:   4.896624
Median:         5.666581
3rd Quartile:   6.724888
Maximum:        11.716121
Length:         1000
Type:           Float64
Number Missing: 0
% Missing:      0.000000

α2
Summary Stats:
Mean:           0.006503
Minimum:        0.004064
1st Quartile:   0.005326
Median:         0.006164
3rd Quartile:   0.007315
Maximum:        0.012744
Length:         1000
Type:           Float64
Number Missing: 0
% Missing:      0.000000

α1
Summary Stats:
Mean:           0.009589
Minimum:        0.005992
1st Quartile:   0.007854
Median:         0.009089
3rd Quartile:   0.010786
Maximum:        0.018791
Length:         1000
Type:           Float64
Number Missing: 0
% Missing:      0.000000
```

In [52]:

```
mode(sort(dfResults,cols = [:R0])[:R0])
```

Out[52]:

```
3.7361113518279803
```

In [53]:

```
describe(sort(dfResults,cols = [:R0])[26:(end-25),:R0])
```

```
Summary Stats:
Mean:           5.914700
Minimum:        3.996596
1st Quartile:   4.934623
Median:         5.666581
3rd Quartile:   6.652141
Maximum:        9.702388
Length:         950
Type:           Float64
Number Missing: 0
% Missing:      0.000000
```

### Results: Plots¶

In [54]:

```
histogram(R0s,legend=:none,xlab="R0")
```

Out[54]:

### Results: Output for plotting in R¶

In [55]:

```
writetable("output_Julia_75.csv",DataFrame(R0=R0s,a1=α1s,a2=α2s,v=vs))
```
