# Supplementary material for: Uncertainty and sensitivity analysis of the basic reproduction number of diphtheria: a case study of a Rohingya refugee camp in Bangladesh, November–December 2017
Source: PeerJ. 2018 Apr 2;6:e4583. doi: 10.7717/peerj.4583 (PMC5885970; doi:10.7717/peerj.4583)
Supplement: Supplemental Information 3 — Julia code of the estimation of the basic reproduction number (creating figures). [file peerj-06-4583-s003.html]

20180227\_Plotting in R


In [176]:

```
libraries = c("distr","readxl","dplyr","magrittr","tidyr","ggplot2","gridExtra","RColorBrewer","reshape2") 
for(x in libraries) {
    library(x,character.only=TRUE,warn.conflicts=FALSE) }

windowsFonts(Times = windowsFont("Times New Roman"))

'%&%' = function(x,y)paste0(x,y)
```

In [177]:

```
filename = "../../data/Data.xlsx"

# load data
read_excel(filename, sheet = 1) %>% ncol -> nclmns
read_excel(filename, sheet = 1, col_types = c("date",rep("numeric",nclmns-1))) %>%
  mutate(Date=as.Date(Date)) %>% mutate(t=1:n()) -> Data.delay  

incd = Data.delay[1:(nrow(Data.delay)-5),4][[1]]
dates = length(incd) 
gtime = p(Gammad(shape=4,scale=2))(1:dates)-p(Gammad(shape=4,scale=2))(1:dates-1)
qN = 579384 #WHO situation report#7 23Dec2017
```

In [178]:

```
output = as.data.frame(read.csv("output_Julia_50.csv",header=T))
output %>% head
```

| R0 | a1 | a2 | v |
| --- | --- | --- | --- |
| 11.330879 | 0.016094511 | 0.008359434 | 0.5894227 |
| 5.200248 | 0.007386490 | 0.003836517 | 0.1053883 |
| 8.286219 | 0.011769850 | 0.006113211 | 0.4385615 |
| 6.470598 | 0.009190894 | 0.004773731 | 0.2810246 |
| 6.898279 | 0.009798399 | 0.005089236 | 0.3255987 |
| 5.904218 | 0.008386464 | 0.004355879 | 0.2120550 |

In [179]:

```
#values for fit
reneq2 = function(R0,p_vaccinated,p_report1,p_report2){
    cumul.i = c(numeric(dates))
    convn =  c(numeric(dates))
    alph = c(rep(p_report1,34),rep(p_report2,(dates-34)))

    St2 = c(numeric(dates))
    dat = incd[1:dates]
        
    for (t in (2:dates)) {
        y = (1:(t-1))
        convn[t] = alph[t]*sum(gtime[y]*dat[t-y]/(alph[t-y])) 
        St2[t] = (1-p_vaccinated-sum(dat[y]/(alph[y]*qN)))
    }

    R0*St2*convn %>% return
}
```

In [180]:

```
fit = sapply(1:(nrow(output)), function(i){
  reneq2(R0=output$R0[i],
         p_vaccinated=output$v[i],
         p_report1=output$a1[i],
         p_report2=output$a2[i])})
fit = as.data.frame(fit)
colnames(fit) = paste("fit", seq(1000), sep="")
rownames(fit) = paste("time",seq(45), sep="")
daf = as.data.frame(fit)
daf$time = rownames(daf)
mdaf = melt(daf, id.vars="time")
mdaf$fit = as.numeric(gsub("fit", "", mdaf$variable))
mdaf$timen = as.numeric(gsub("time", "", mdaf$time))

edates = 1:dates# add date information
edates = as.Date(edates, origin="2017-11-07")
mdaf$timed=rep(edates)

sortlist= order(mdaf$timed) # reorder by date
mdaf = mdaf[sortlist,] 
mdaf$incd=rep(incd,each=1000)
```

In [181]:

```
options(repr.plot.width=6,repr.plot.height=4.5)

pf = mdaf %>% 
    ggplot(aes(x = timed, y = incd, group=fit))+
    geom_bar(stat = "identity",colour="deepskyblue2",  position = position_dodge(width=0.7))+
    labs(x="time", y="number of new cases")+
    scale_x_date(date_breaks = "1 day",date_labels = "%b %d") +
    coord_cartesian(ylim=c(0,150)) +
    theme_bw(base_family='Times') +
    theme(axis.text=element_text(size=10),
        axis.text.x = element_text(size=9, angle = 90, vjust = 0.5),
        axis.title=element_text(size=12), 
        panel.grid= element_blank())

ggsave(filename="Fig1.tiff", plot=pf, width=180, height=120, units="mm", dpi=300)

pf
```

In [182]:

```
options(repr.plot.width=6,repr.plot.height=4.5)

pf = ggplot(mdaf, aes(x = timed, y = incd, group=fit))+
  geom_bar(stat = "identity",colour="deepskyblue2", position = position_dodge(width=0.7))+
  geom_point(aes(x=timed, y=value, group=fit), size=1.0, colour="deepskyblue4")+
  labs(x="time", y="number of new cases")+
  theme_bw(base_family='Times')+
  theme(axis.text=element_text(size=10, colour="black"),
        axis.title=element_text(size=12), 
        panel.grid= element_blank())

pf
```

In [183]:

```
options(repr.plot.width=7,repr.plot.height=6)

#2D scatterplot; 
#R0 vs V
Rvp = output %>% ggplot(aes(x=v,y=R0))+
    geom_point(colour="deepskyblue4", size=1)+
    xlab(expression(italic(v))) + 
    ylab(expression(italic(R)[0])) +
    theme_bw(base_family='Times')+
    theme(axis.text=element_text(size=10),
          axis.title=element_text(size=12),
          panel.grid= element_blank())
         
#R0 vs alpha1
Ra1p = output %>% ggplot(aes(x=a1,y=R0))+
    geom_point(colour="deepskyblue4", size=1)+
    xlab(expression(italic(a)[1])) + 
    ylab(expression(italic(R)[0])) +
    theme_bw(base_family='Times') +
    theme(axis.text=element_text(size=10),axis.title=element_text(size=12),panel.grid= element_blank())+
    scale_x_continuous(labels=function(x) sprintf("%.3f", x))
                       
#R0 vs alpha2
Ra2p = output %>% ggplot(aes(x=a2,y=R0))+
    geom_point(colour="deepskyblue4", size=1)+
    xlab(expression(italic(a)[2])) + 
    ylab(expression(italic(R)[0])) +
    theme_bw(base_family='Times')+
    theme(axis.text=element_text(size=10),axis.title=element_text(size=12),panel.grid= element_blank())
         
# 2D figures
twoD_fig = grid.arrange(Rvp,Ra1p,Ra2p,pf,nrow=2)
ggsave(filename="Fig2.tiff", plot=twoD_fig, width=180, height=120, units="mm", dpi=300)
```

In [184]:

```
options(repr.plot.width=8,repr.plot.height=4)

#Histogram for one dimentionals
output = as.data.frame(output)

nbins = 40
ymax = 80

#R0
R0pic = ggplot(output, aes(x=R0)) +
    geom_histogram(colour="black", fill="deepskyblue4", bins=nbins) +
    ylab("frequency") +
    xlab(expression(italic(R)[0])) +
    theme_bw(base_family='Times') +
    theme(axis.text=element_text(size=10),
          axis.title.x=element_text(size=14),
          panel.grid= element_blank()) +
    scale_x_continuous(labels=function(x) sprintf("%.1f", x)) +
    scale_y_continuous(breaks=seq(0, ymax, by=20),limits = c(0, ymax))

#alpha1
a1pic = ggplot(output, aes(x=a1))+
    geom_histogram(colour="black", fill="deepskyblue4", bins=nbins)+
    xlab(expression(italic(a)[1])) +
    scale_x_continuous(labels=function(x) sprintf("%.3f", x))+
    scale_y_continuous(breaks=seq(0, ymax, by=20),limits = c(0, ymax)) +
    theme_bw(base_family='Times') +
    theme(axis.text=element_text(size=10),
          axis.title.x=element_text(size=14),
          axis.title.y = element_blank(), 
          panel.grid= element_blank())

#alpha2
a2pic = ggplot(output, aes(x=a2))+
    geom_histogram(colour="black", fill="deepskyblue4", bins=nbins, position = position_dodge(width=0.7))+
    xlab(expression(italic(a)[2])) +
    theme_bw(base_family='Times')+
    theme(axis.text=element_text(size=10),
          axis.title.x=element_text(size=14),
          axis.title.y = element_blank(), 
          panel.grid= element_blank())+
    scale_y_continuous(breaks = seq(0, ymax, by=20),limits = c(0, ymax))+ 
    scale_x_continuous(labels=function(x) sprintf("%.3f", x))
                       
sing = grid.arrange(R0pic,a1pic,a2pic,nrow=1)
ggsave(filename="Fig3.tiff", plot=sing, width=180, height=60, units="mm", dpi=300)
                       
sing
```

```
TableGrob (1 x 3) "arrange": 3 grobs
  z     cells    name           grob
1 1 (1-1,1-1) arrange gtable[layout]
2 2 (1-1,2-2) arrange gtable[layout]
3 3 (1-1,3-3) arrange gtable[layout]
```

# Sensitivity analysis (Fig 4)¶

In [185]:

```
data.frame(R0 = read.csv("output_Julia_50.csv",header=T)$R0, CV = "50%") %>%
    rbind(data.frame(R0 = read.csv("output_Julia_25.csv",header=T)$R0, CV = "25%")) %>%
    rbind(data.frame(R0 = read.csv("output_Julia_75.csv",header=T)$R0, CV = "75%")) -> dfSensitivity

dfSensitivity %>% head
```

| R0 | CV |
| --- | --- |
| 11.330879 | 50% |
| 5.200248 | 50% |
| 8.286219 | 50% |
| 6.470598 | 50% |
| 6.898279 | 50% |
| 5.904218 | 50% |

In [192]:

```
options(repr.plot.width=4,repr.plot.height=4)

dfSensitivity %>%
    ggplot(aes(x = CV, y = R0)) +
    geom_boxplot(colour="deepskyblue4", fill="deepskyblue2",outlier.shape = NA) +
    scale_x_discrete(limits=c("25%","50%","75%")) +
    ylab(expression(italic(R)[0])) +
    coord_cartesian(ylim=c(4,16)) +
    theme_bw(base_family='Times') +
    theme(axis.text=element_text(size=10),
          panel.grid= element_blank()) -> plt_cv

ggsave(filename="Fig4.tiff", plot=plt_cv, width=60, height=80, units="mm", dpi=300)

plt_cv
```
